# Off-label use of orphan medicinal products: a Belgian qualitative study

**DOI:** 10.1186/s13023-016-0507-y

**Published:** 2016-10-28

**Authors:** Marc Dooms, David Cassiman, Steven Simoens

**Affiliations:** 1 University Hospitals Leuven, Centre of Clinical Pharmacology, Herestraat 49, 3000 Leuven, Belgium; 2 Division of Gastroenterology-Hepatology, University Hospitals Leuven, Herestraat 49, 3000 Leuven, Belgium; 3 KU Leuven Department of Pharmaceutical and Pharmacological Sciences, Herestraat 49, PO box 521, 3000 Leuven, Belgium

**Keywords:** Off-label use, Unlicensed use, Orphan medicinal products, Rare diseases, Safety, Efficacy

## Abstract

**Background:**

Off-label use of (orphan) medicinal products for (rare) diseases is quite common but not underpinned by clinical studies to confirm efficacy and safety. No risk-analyses by regulatory agencies are carried out. The objective of this study was to map off-label use of orphan medicinal products in Belgium in terms of attitude towards off-label prescribing, factors influencing off-label prescribing, disclosure of information towards the patient, reporting of off-label use, risks and consequences. Most of the EMA authorized orphan drugs are fully reimbursed in Belgium under well-defined circumstances. Moreover, a “Special Solidarity Fund” takes care of some specific cases eventually prescribed off-label.

**Methods:**

Semi-structured interviews with seven physicians with expertise in the treatment with and six experts in the reimbursement of orphan medicinal products in Belgium. This task was performed by five last-year pharmacy students after having studied profoundly the medical literature around off-label prescribing. They had no previous contact with the participants.

**Results:**

Most participants do agree with the off-label use if the medicinal product is quite safe and well-tolerated, if the on-label indication is rather general and when all other options have failed in some specific, evidence-based indications, especially in children. Before starting off-label use, the patient/family needs to be fully and clearly informed. The treatment is not reimbursed but sometimes sponsored by the company or by charity funds. Reporting of the outcome is necessary to avoid losing valuable information. The prescriber is responsible and can be held accountable.

**Conclusions:**

While there is support from physicians and reimbursement experts, there is also concern in case of off-label use, mainly for reasons of patient safety especially when medicinal products are prescribed off-label in the absence of medical or scientific justification and driven by cost-containment motives.

## Background

Off-label use of a medicinal product entails the intentional use of the medicinal product for any indication, population, dosage, administration route or treatment duration other than that approved by a country’s regulatory authority [1]. For the most part, off-label use of medicinal products is not underpinned by rigorous clinical and non-clinical studies necessary to confirm quality, efficacy and safety [2]. Off-label use of medicinal products is quite common; 21 % of all prescription medicinal product use is supposedly off-label. In some disease areas off-label use can be as high as 83 % [1]. Only about 30 % of off-label prescribing is supported by adequate scientific data [2]. According to Gupta et al. [2], off-label use is more common if standard treatments fail or are non-existent.

In the European Union, rare diseases are defined as life-threatening or chronically debilitating diseases, which occur at such a low prevalence (5 in 10,000 EU citizens) that special initiatives are needed to address them [3]. It is estimated that there are currently between 5000 and 7000 rare diseases. Orphan medicinal products (OMPs) are intended to diagnose, prevent, or treat rare diseases. With only 118 orphan medicinal products on the European market at the beginning of 2016, only a small part of the treatment need for rare diseases is covered [4]. These OMPs are sometimes used off-label for rare disease patients in case the ‘label’ imposes certain restrictions; for example, some OMPs are used in pediatric populations regardless of age restrictions [5]. Off-label use of OMPs in more common indications also occurs; for example epoetin alfa was originally registered in the US as an orphan medicinal product to treat anemia in end-stage renal disease patients but its use soon became more widespread [6]. Finally, rare disease patients are also often being treated (off-label) using other ‘non-orphan’ medicinal products. Rare disease patients appear to be exposed to off-label use more often than patients with more common diseases, partly due to the limited number of patients [7]. Indeed, up to 90 % of all medicinal product use for rare diseases is off-label according to Liang et al. [1].

Yet, there are risks associated with off-label use; clinical evidence demonstrating quality, safety and efficacy is often lacking and no risk-analysis by regulatory agencies is carried out [2, 8]. Although patients with a life-threatening disease are likely to be more risk-acceptant, EU legislation states that “patients suffering from rare conditions should be entitled to the same quality of treatment as other patients” [3]. As such, off-label use of medicinal products for rare disease patients has been compared to a double-edged sword; on the one hand it might be a last resort for patients in unique life-threatening situations, on the other hand, it also exposes them to risks and experimentation [2].

Even though the extent of off-label use of OMPs appears to be significant; Kesselheim et al. found that in a sample of four top-selling orphan medicinal products, three orphan medicinal products were used more commonly for non-orphan indications; the reasons behind off-label prescribing of medicinal products for rare disease patients are yet to be fully understood [6]. Therefore, the aim of this study was to map off-label use of OMPs in Belgium in terms of attitude towards off-label prescribing, factors influencing off-label prescribing, disclosure of information towards the patient, reporting of off-label use, risks and consequences. This study is based on the work of five pharmacy students in the context of their Master theses at KU Leuven [9]. Mapping out these issues is a first step in achieving a more systematic approach for appropriate off-label prescribing.

While there is support for off-label use in specific circumstances, there is a growing trend towards promoting the prescription of medicinal products off-label without medical justification but for other motives such as cost-containment and economic reasons [10, 11]. There is a risk that these trends would compromise patient safety. EU Member States are passing legislation, guidelines or establishing practices promoting off-label use mainly to reduce healthcare spending - for instance in Italy, France and Denmark [12]. These practices create unnecessary and avoidable risks for patients, often without their consent. This view is supported by the European Court of Justice, which has ruled that patient safety must always prevail against any economic motives. The Court of Justice confirmed this principle in its 2012 judgment in the case Commission vs. Poland (C-185/10). The Court concluded that Poland had been misusing a rule that allows the use of medicinal products for named patients and had been importing medicinal products that were not authorized there, even though authorized equivalents were available. The Court decided that the Polish law infringed upon the marketing authorization requirements.

## Methods

Semi-structured interviews were used as they enable the interviewer to elaborate on specific aspects or insights of the interviewee.

### Participants

University Hospitals Leuven (Belgium) is a large tertiary referral centre with broad experience in all rare disease areas and orphan medicinal product use. A national multidisciplinary consultation for rare diseases and several national and international reference networks operate in this hospital such as for haemophilia, cystic fibrosis, spina bifida, pulmonary arterial hypertension, amyotrophic lateral sclerosis, cystinosis, epidermolysis bullosa, ichthyosis and neurofibromatosis. A total of 18 physicians treating rare diseases and/or using orphan medicinal products from University Hospitals Leuven and 9 experts involved with (orphan) medicinal product policy and/or reimbursement procedures in Belgium were contacted by e-mail to participate in this study. A reminder was sent after 1 week. Respondents were identified through selective sampling by the authors. Respondents who indicated to be willing to participate in the interviews were contacted by e-mail for further arrangements concerning time and place of the interview.

### Interviews

A semi-structured interview guide was drafted based on topics derived from a literature review which was undertaken by the group of pharmacy students. Two experts were given the opportunity to comment on the content validity of this semi-structured interview guide, after which it was adapted according to their comments. A pilot interview took place on September 13^th^ 2013. Appropriate approval was obtained from University Hospitals Leuven Medical Ethics Committee on July 12^th^ 2013 (approval n° S55673).

All interviews were conducted between September 13^th^ 2013 and November 7^th^ 2013 by the group of five pharmacy students supervised by SS, MD and Eline Picavet. All respondents signed an informed consent form in duplicate. One copy of each form was kept by the researchers. All respondents participated voluntarily and were not remunerated. Anonymity of the participants and confidentiality of the answers were guaranteed. The interviews were audio-recorded and transcribed *verbatim*.

### Data analysis

The interviews were analysed using the software QSR NVivo 10 according to the five stages of the framework analysis: (1) familiarization (reading of the transcripts and notes, listening to the digital recordings); (2) identifying a framework; (3) indexing (application of the framework to the data); (4) charting; and (5) mapping and interpreting [13]. Selected quotes of the interviewees were translated as accurately as possible.

## Results

A total of seven physicians treating rare diseases and/or using orphan medicinal products from University Hospitals Leuven (Belgium) and six experts involved with (orphan) medicinal product policy and/or reimbursement procedures in Belgium participated in the study. All participants were coded according to their background; i.e. physicians were assigned codes starting with an A and policy experts were assigned codes starting with a B.

### Attitude towards off-label prescribing of OMPs

Most policy makers are not firmly in favor or against off-label prescribing. Some only consider it appropriate within the framework of a clinical study and if there is no “on-label” alternative available.

*“I’m against under the current conditions, data are hardly being collected.” (* ***BNY31102*** *)*

*“I think it’s too simplistic to be “against” off-label use, but I do think that it has to happen in the context of study projects as much as possible.” (* ***BES14101*** *)*

*“Of course you have to check first whether there is no approved medicinal product for that indication. But if there isn’t, then off-label prescribing should have to be possible.” (* ***BNS11101*** *)*

*“I think OMPs need to be prioritized here, because they treat rare diseases. The clinical evidence will always be limited, but if you have a proof of concept, an idea of how a medicinal product works, then I agree that it should be available for patients as quickly as possible. It would even be unethical not to!.” (* ***BTY14102*** *)*

### Factors influencing off-label prescribing

According to the interviewees several factors influence the choice to prescribe an off-label treatment.

#### Safety and tolerability

The barrier to prescribe a medicinal product off-label is lower if the medicinal product is considered safe and well-tolerated.

*“The most defining factor is the safety of the medicinal product and whether or not it causes severe side effects. You don’t want to make a patient even sicker than he already is.” (* ***AET13091A*** *)*

*“Once you go off-label, I think safety is the most important thing. Safety and tolerability. (…) Medicinal products with a good safety profile have a better chance of getting prescribed off-label.” (* ***ANS02101L*** *)*

#### Effectiveness

All stakeholders agree that, although the evidence on the effectiveness of a medicinal product may be limited, treatment decisions should still be evidence-based. Physicians keep up to date by following publications within their area of expertise. New findings are also discussed with national and international colleagues. Peer-to-peer consulting is considered a crucial aspect, even though the ultimate decision to start a new off-label treatment is personal.

*“You hardly ever have guarantees about the effectiveness of a medicinal product in the off-label indications. (…) But you need to have some sort of scientific indication that it actually works. You can’t just prescribe medicinal products and hope that they will have an effect.” (* ***AET13091A*** *)*

*“I always consider: has this medicinal product been tested in randomized studies? And I try to discuss it with my assistants: why are we choosing this medicinal product?” (* ***AES14103A*** *)*

*“There is a forum for questions about effectiveness and cost-effectiveness of medicinal products used for metabolic diseases. You can even add your own questions. (…) That’s how you get feedback from physicians worldwide. Sometimes is somewhat subjective or not always based on objective criteria from the literature, but along the way you learn to distinguish between what is relevant and what is not.” (* ***AES14103A*** *)*

#### Mechanism of action

Medicinal products with a more general and broad mechanism of action, like some oncology medicinal products, are more likely to be prescribed off-label compared to other more targeted medicinal products such as enzyme replacement therapies.

*“These medicinal products [enzyme replacement therapies] are used exclusively for very specific indications, so I guess they are not used for any other indications.” (* ***ANS10101A*** *)*

*“You can of course, based on the mechanism of action of a medicinal product presume that it will probably also be effective for a similar disease and just try it. That is how it is often done.” (* ***ANS10101A*** *)*

#### Place in clinical practice

Off-label prescribing is seldom applied as first-line therapy. It is considered when all other options have failed.

*“At the end of what is feasible, or what is reimbursed, or what is “standard”, then you resort to off-label prescribing.”* *(* ***ANS02101L*** *)*

#### Disease indication

Off-label prescribing is more frequent in some specific indications

*“There are 1001 medicinal products to treat heart arrhythmia. If product A fails, then try product B. And all these are registered and reimbursed. There is no such thing in hemato oncology! Additionally, medicinal products in hemato oncology are mostly expensive, monoclonal antibodies and small molecules. Off-label use is a lot more frequent in hemato oncology.” (* ***ANS02101L*** *)*

#### Patient population

A final determining factor is the patient population. Children are subjected to off-label use more often than adults because not all medicinal products are registered for paediatric use.

*“Yes, in neonatology intensive care, I think it will be up to 80 or 90 % off-label. Almost every leaflet states that the medicinal product can’t be used in children.” (* ***AET07111C&N*** *)*

### Disclosure of information towards the patient or family

Before starting an off-label treatment, the patient needs to be informed about the working mechanism and risks associated with the treatment. According to the interviewees it’s important that patients are aware that the indication is not officially registered and that the clinical evidence is limited.

*“We basically don’t have a choice, if it isn’t a registered indication. It’s something between an experiment and standard common practice, so we ask for permission.” (* ***AET1309A*** *)*

*“The decision to prescribe an off-label medicinal product is made in consultation with the patient.” (* ***BNS02101*** *)*

*“Yes, we need informed consent. A patient HAS to say: ‘I share responsibility’. Provided of course that the patient has been informed correctly.” (* ***BTY14102*** *)*

The information provided to the patient regarding the off-label treatment must be clear and comprehensible. All too often, specialist jargon is used, causing patients to given their consent based on unclear information. In general, patients and their family do not oppose off-label use.

*“The specific jargon is sometimes incomprehensible to the patient, so it needs to be explained very clearly. And I’m not saying that the information should be trivialized or popularized, but still… (…) Patients are sometimes desperately looking for a last remedy, so it’s not uncommon that they agree too easily or too quickly.” (* ***BTY14102*** *)*

*“Also, the physician needs to have the information himself! I think there is a role there for the industry. They know their medicinal product, they can say ‘these are the limitations’.” (* ***BTY14102*** *)*

*“If there aren’t any alternatives, and the patient is very sick, he is likely to be happy with any alternative. Most patients don’t oppose.” (* ***AET13091A*** *)*

*“We mustn’t forget that, these days, patients are well informed! They scan the internet, find a product and suggest it to ME. And if it is not available, we try to get it.” (* ***ANS02101L*** *)*

Physicians inform patients that there is a chance that the treatment is stopped because of lack of effectiveness or because of financial reasons. To avoid disappointments, a timeframe in which an effect should be noticeable is agreed upon in advance.

*“At a certain point, we stopped using the medicinal product and the family agreed. They also had the impression that that was for the best. We had discussed this on beforehand.” (* ***AES14103A*** *)*

*“If it doesn’t have the desired effect after a certain time, then you stop the treatment. But we have this in mind in the beginning. For example, after four courses of treatment, if it doesn’t have an effect, we stop trying.” (* ***ANS02101L*** *)*

### Financing and budget for reimbursement

Off-label use is not officially reimbursed.

*“Reimbursement is limited to the indications mentioned in the SpC. Sometimes it is even more limited, but never more broad. So off-label use is not reimbursed. But the physician can prescribe it for another reimbursed indication of course.” (* ***BNS11101*** *)*

*“I think we should be very careful with reimbursement of off-label use. If too much is ‘allowed’, then companies no longer will be motivated to apply for new indications and to perform their own studies. But sometimes, there is no other choice, just to help patients!” (* ***BES14101*** *)*

In practice, off-label use is avoided in case there would be an associated financial burden for patients or family. In some cases the company offers the product for free. If not, financial support can be offered by special governmental funds (i.e. the Exceptional Solidarity Fund in Belgium) or (charity) funds from hospitals.

*“The patient feels no financial burden, because we make sure of it! We want to avoid that people do crazy things, like selling their house, to pay for a treatment that won’t necessarily benefit them.” (* ***ANS0201L*** *)*

*“We mostly try to get the product from the company. Other options are national or internal solidarity funds. But these are exceptions! The odds of getting a positive response there are very small.” (* ***ANS02101L*** *)*

*“We [at the hospital] have an internal solidarity fund, but there is hardly any money available. I think if we were to treat one patient with an enzyme replacement therapy, well that would drain the fund completely for the rest of the year.” (* ***AET13091A*** *)*

Finally, reimbursement can also be obtained by willfully falsifying medical certificates.

*“Off-label use is not reimbursed, unless we claim that the product will be used to treat a reimbursed indication. For example if one subgroup is reimbursed, but the other is not, then you can prescribe everything for that first subgroup. But if you get caught… you can pretend you made a mistake once, but not ten times! I’m not going to deny that we sometimes bend the rules like that.” (* ***ANS02101L*** *)*

*“Sometimes companies send delegates to physicians to explain how they can by-pass the law, which boxes to check on the application form. Of course it’s cheaper for them! And in the end, they are not responsible; when problems arise, well they say ‘it’s off-label use’.” (* ***BES14101*** *)*

The total share of the entire pharmaceutical budget allocated to off-label orphan medicinal product use is not easily determined. Interviewees disagree on whether or not this share is high or low.

*“It can be high, I would guess up to 50 % of the total orphan medicinal product budget. But there is no way to determine it, it’s a speculation.” (* ***BES14101*** *)*

*“You could map it, but that would be very difficult! I guess because orphan medicinal products are only used for a limited number of patients the off-label budget will be limited.” (* ***BTY14102*** *)*

### Reporting of off-label use

In practice, off-label use is seldom reported in the literature. Publishing individual case studies is not considered worth the effort. Nevertheless, experts agree that reporting of off-label use is necessary to avoid losing valuable information. A European database is considered an option.

*“Well I have to admit that the pressure to publish positive results is bigger than to publish bad results. Bad results do not get published, period.” (* ***ANS02101L*** *)*

*“Case reports are just cast reports with little scientific value. (…) I’m more in favor of organizing clinical studies or registers or systems in which results are somewhat grouped.” (* ***BES14101*** *)*

*“Yes, I think we have to report it for these rare conditions simply because the medicinal products are so expensive. (…) It would even be best to organize it on a European level. For example, everybody using an orphan medicinal product off-label, registering that use in a European database.” (* ***AES14103A*** *)*

There is no agreement on whether or not such database should be anonymous. Anonymity protects the physician, so that the barrier for reporting negative experiences is lowered.

*“Anonymizing, that’s an option. It can possibly help to also publish negative experiences.” (* ***BNS11101*** *)*

*“That the physician is anonymous? Well yes of course! (…) But, if someone else wants more information, how would that work? Why would physicians want to remain anonymous? I can imagine that all of us are trying to achieve improvements in medical science, and that you stand by your own decisions and take responsibility for them.” (* ***AET13091A*** *)*

*“Of course it’s useful! You want to spare others the mistakes that you made. Well it doesn’t necessarily even have to be a failure, but just a ‘lack of effect’, I think it’s also useful to report that. So others won’t try the same thing.” (* ***AET13091A*** *)*

*“Absolutely, those negative experiences give you a much bigger picture! Nowadays everybody has a feeling of ‘wow this works well off-label’, but in fact, you don’t know about all the failed attempts.” (* ***ANS02101L*** *)*

A recurring concern is the amount of paperwork involved in reporting off-label use. A simple form of reporting should therefore be considered. Some highlight the need for a regulatory framework.

*“We simply don’t have the time to do all that publishing and reporting.” (* ***AEY11101L*** *)*

*“This database, it won’t be easy to implement. At the moment there is no legal framework, and I think that is needed first.” (* ***BNS11101*** *)*

Some physicians already use disease specific registers to keep track of off-label use.

*“For some conditions, or groups of conditions, there are international registers. I try to participate in those. It gives me a lot of information! If everyone has five patients, that’s not informative, but all together, well there might be 500 and then you learn something from it.” (* ***AET13091A*** *)*

### Risks and consequences

#### Therapeutic failure

The most important risk, according to the interviewees, is failure of the off-label treatment, either due to failure to achieve the desired effect or due to the occurrence of serious adverse events.

*“Based on the literature we thought, we have to do something and this looks promising. In practice, it didn’t play out well.” (* ***AES14103A*** *)*

*“It isn’t always 100 % clear what the effects and the adverse events will be.” (* ***AET13091A*** *)*

#### Accountability

The prescriber is responsible for off-label prescribing and can be held accountable. Most physicians do not consider this a big risk. None of the interviewed physicians had negative experiences.

*“A physician has therapeutic freedom and can prescribe off-label. But it is a risk! Companies and pharmacists will not cover the physician’s back if anything goes wrong.” (* ***BNS11101*** *)*

*“A risk? Not really. We prescribe so many strange medicinal products.” (* ***AEY11101L*** *)*

*“It is not a risk, it’s based on the literature and you discuss it with patient beforehand. Everybody knows what the risks are. In my opinion, the only risk is the reimbursement of the product.” (* ***ANS02101L*** *)*

#### Funding problems

Another risk is the lack of a posteriori financing. In most cases, a treatment is started before reimbursement or funding through alternative channels has been approved. Treatment can be stopped abruptly if these fail.

#### Incentives for future development

Pharmaceutical companies may wish to extend the indication of an orphan medicinal product. However, the risk of downward pressure on the price and the efforts needed to collect data are important hurdles.

*“But I think you have to be careful to extend an indication, because that’s a practice that companies strive for. They put a product on the market in a small niche market, but their actual target group is much wider to be able to make profits.” (* ***AES14103A*** *)*

*“Why don’t they [pharmaceutical companies] file for additional indications? Because when that happens, they have to lower the price! It’s an odd situation of course.” (* ***BNS02101*** *)*

*“Companies are not interested. Why would they be? Their product is the only one on the market… If they change the application, then it has to be based on clinical studies. And the evidence is already there, because physicians are collecting it. Is it worthwhile to trouble children again?? There is a lot more behind it than the motivation of the company.“ (* ***AET07111C&N*** *)*

*“It is not easy to collect data in these very small patient populations! This implies that the number of phase II and III clinical studies is limited, and that the duration of these studies may be limited.” (* ***BNS02101*** *)*

*“From a societal point of view it is sometimes frustrating that we can’t force a company to extend an indication.” (* ***BES14101*** *)*

### Future perspectives

According to some policy makers, off-label prescribing should be limited to expert centers.

*“I don’t think everybody should be allowed to prescribe everything. We trust expert centers, we determine their expertise, they fulfill all our conditions. Then they also should be able to prescribe off-label in a responsible way.” (* ***BNS02101*** *)*

## Discussion

This study has mapped off-label use of OMPs in terms of attitude towards off-label prescribing, factors influencing off-label prescribing, disclosure of information towards the patient, reporting of off-label use, risks and consequences in Belgium. Our results show that off-label prescribing of OMPs is a common practice, especially when alternative treatments are lacking. A legal framework should put all parties involved at ease.

It is our opinion that off-label prescription should only occur after individual assessment by the treating physician of the needs of the individual patient. Physicians need to be given the freedom to uphold their pledge towards their patients to act ethically and put the patient’s interest first. They should be supported by the public bodies and authorities responsible for the approval and usage of medicines whose role is to protect public health. Patients should be informed about the off-label use and the consequences. Lately, a new trend to tackle this issue appeared. Novartis brought the same active ingredient (everolimus) in the same dose and the same pharmaceutical form on the market but under two different names: Afinitor® for use in oncology and Votubia® for the treatment of tuberous sclerosis. Boehringer Ingelheim did the same with nintedanib: Ofev® with interstitial pulmonary fibrosis on the label and Vargatef® with an oncology label.

Pharmaceutical companies are not allowed to mention potential off-label uses for their already marketed products. However, they can ask for a second or further medical use claim or an orphan designation when the indication is rare. Other companies repurpose off-patent active ingredients in another medication for an already known off-label use: ibuprofen injection as Pedea® and 3,4-diaminopyridine capsules as Firdapse®. When a medicinal product is taken from the market by the pharmaceutical company as the on-label indications become outdated for example, off-label users lose their treatment: Calcort® (deflazacort) for Duchenne muscular dystrophy and Mexitil® (mexilitine) for rare forms of myotonias. Payers sometimes reimburse off-label use but mostly they wish the companies to prove safety and efficacy before they consider reimbursement: thalidomide is reimbursed in several rare dermatologic indications in Belgium such as prurigo nodularis, Osler-Weber-Rendu and Behçet disease.

In the academic literature, there is a tendency to report only positive experiences with off-label products rather than recording adverse events [14–18]. Literature can therefore be unbalanced and an effort should be made to publish articles and reports of adverse events linked to the off-label use of a product. It is crucial for patients and physicians to report adverse events and outcomes as accurately as possible with a view to install guidelines for the potential treatment of these conditions with OMPs. Furthermore, the use of patient registries should be encouraged. The EudraVigilance platform, the European data processing network and management system for reporting and evaluating suspected adverse reactions could be used to gather better data on adverse effects by patients as well as medical professionals. Recently, pharmaceutical companies need to register pharmacovigilance even for off-label use of their pharmaceutical products.

This qualitative study mapped out off-label use of OMPs through in-depth interviews with both policy makers as well as physicians. However, this study was subject to some limitations. The outcome of an interview is dependent on the experiences and expertise of both the interviewer and the interviewee on the topic. Moreover, the use of a pre-defined, semi-structured interview guide can limit the course of the interview and the issues discussed. Future research should not only include the experiences of physicians in a university hospital (which acts as a referral centre for patients with rare diseases), but also be extended to physicians in general hospitals.

## Conclusions

This study showed that off-label use can be useful especially in the treatment of patients with rare diseases. However, we need some guidelines [12] to protect patients and practitioners, and to avoid cost-containment practices compromising patient safety. Therefore, we propose that off-label use of medicinal products should only occur if the following criteria are met:

**Table:** 

1. Presence of a severe, life-impairing or life-threatening condition; 2. Absence of authorized treatment or repeated treatment failure; 3. Absence of alternative treatments authorized for the condition; 4. The off-label use is supported by strong evidence in scientific literature; 5. The patient has been educated and has given his or her informed consent; 6. Presence of established reporting routes for adverse events and linked to off-label use.	

## Abbreviations

EU: European Union
NVivo: Software that supports qualitative and mixed methods research (SciencePlus.nl)
OMP: Orphan medicinal product
SpC: Summary of product characteristics
